# Type III Collagen Glomerulopathy Presenting With Divergent Histopathological Patterns: Report of Two Adult Cases and Diagnostic Pitfalls

**DOI:** 10.1155/crin/6022775

**Published:** 2026-09-22

**Authors:** Fatemeh Nili, Alireza Abdollahi, Parisa Arabmohammadi, Samaneh Salarvand

**Affiliations:** ^1^ Department of Pathology, School of Medicine, IKHC, Tehran University of Medical Sciences, Tehran, Iran, tums.ac.ir

**Keywords:** electron microscopy, glomerulopathy, hematuria, kidney biopsy, proteinuria, Type III collagen

## Abstract

Type III collagen glomerulopathy (collagenofibrotic glomerulopathy) is a rare idiopathic renal disorder. It is characterized by abnormal deposition of Type III collagen fibrils within the mesangial and subendothelial compartments of glomeruli. Due to its nonspecific clinical presentation and histopathologic overlap with more common glomerular diseases, it is frequently underdiagnosed unless electron microscopy is performed. We report two adult male patients who presented with progressive renal dysfunction, proteinuria, hematuria, and hypertension. In both cases, initial renal biopsies were misleading. The first patient received a diagnosis of tubulointerstitial nephritis; the second was diagnosed with focal segmental glomerulosclerosis (FSGS). Repeat kidney biopsies revealed mesangial expansion, glomerular basement membrane thickening, and variable degrees of chronic glomerulosclerosis on light microscopy. Immunofluorescence was negative or nonspecific in both cases, with no significant immune complex deposition. Definitive diagnosis was established by electron microscopy, which demonstrated characteristic curvilinear collagen fibrils, approximately 60 nm in diameter, within the mesangial and subendothelial spaces, without evidence of immune complex deposits. The two cases represented different stages of disease. One showed relatively preserved glomerular architecture consistent with earlier‐stage involvement, while the other exhibited advanced diffuse glomerulosclerosis and severe tubulointerstitial fibrosis. Together, they illustrate the broad morphologic spectrum of this entity. These cases highlight the diagnostic challenge posed by Type III collagen glomerulopathy and its frequent misclassification as other glomerular diseases, particularly FSGS and membranoproliferative patterns. Our findings underscore the essential role of electron microscopy in reaching a definitive diagnosis. Given how often this condition is missed, it may be underrecognized rather than truly rare. Early and accurate diagnosis is critical to avoid mismanagement in patients with unexplained proteinuric kidney disease.

## 1. Introduction

Type III collagen glomerulopathy, also called collagenofibrotic glomerulopathy, is a rare idiopathic glomerular disease [1]. It is characterized by abnormal buildup of Type III collagen in the mesangial and subendothelial spaces of glomerular capillaries. Arakawa et al. first described this condition in 1979. It accounts for about 0.1% of all renal biopsies, with only around 100 cases reported globally [2, 3]. Most early cases were reported from Japan [4]; however, cases have now been found in the United States, Europe, India, South America, and China [5–7].

The pathogenesis of Type III collagen glomerulopathy is still unclear. The exact site and mechanism of excessive Type III collagen production are unknown, although intrinsic renal defects have been proposed based on the absence of disease recurrence in renal allografts [2, 8]. Most adult patients have no family history. In contrast, children with this disease often demonstrate familial nephropathy patterns. This suggests autosomal recessive inheritance in some families. A few cases have been linked to mutations in complement factor H (CFH) and atypical hemolytic uremic syndrome [9, 10].

Clinically, patients develop proteinuria, often in the nephrotic range, accompanied by edema [6, 7]. Approximately 20% of cases have microscopic hematuria. Many patients also have high blood pressure [5, 8]. The disease usually affects young adults between 20 and 40 years old. However, cases have been reported from infancy to 79 years, with equal gender distribution [5, 11].

Diagnosis requires kidney biopsy. Light microscopy shows enlarged, lobular glomeruli without inflammation, with capillary wall thickening and mesangial expansion by pale eosinophilic material [8]. Definitive diagnosis requires electron microscopy (EM) demonstrating fibrillary collagen deposits with curved fibrils showing approximately 60‐nm periodicity in the mesangium and subendothelial space [6]. Immunohistochemistry (IHC) confirms the diagnosis with positive staining for Type III collagen in the mesangium and capillary loops. Immunofluorescence (IF) is typically negative, showing no significant immune complex deposition [7]. Clinical exclusion of nail‐patella syndrome is essential for definitive diagnosis [10]. Herein, we present two cases of Type III collagen glomerulopathy with characteristic clinical, laboratory, and pathological findings.

## 2. Case Presentation

### 2.1. Case 1

A 42‐year‐old male with a known history of hypertension presented with a 6‐month progressive decline in renal function. He developed rising serum creatinine, proteinuria, and microscopic hematuria. Renal ultrasonography demonstrated slightly small kidneys with increased cortical echogenicity. Laboratory investigations revealed serum creatinine of 2.5 mg/dL and blood urea of 140 mg/dL. An initial renal biopsy had been performed, which showed tubulointerstitial nephritis with foci of acute tubular injury, but no definitive glomerular pathology was identified. Given the persistent renal dysfunction and lack of a clear diagnosis, a repeat renal biopsy was undertaken.

**FIGURE 1:**
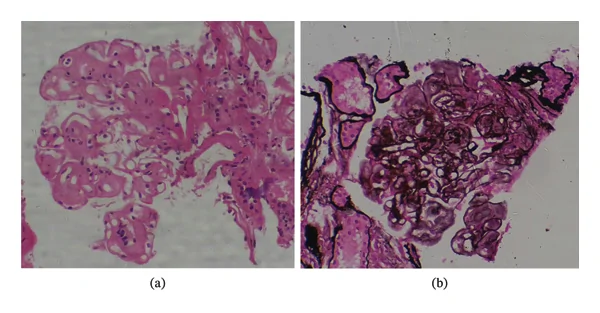
(a) Mesangial expansion by a homogeneous, pale material and segmental hypercellularity in H&E stain, x800 magnification. (b) Double contour formation due to pale material deposition, Jones silver stain, x800 magnification.

Light microscopy of the repeat biopsy revealed needle‐shaped corticomedullary tissue containing up to 2 glomeruli on serial sections. Both glomeruli showed no sclerosis but demonstrated global mesangial expansion by homogeneous pale material and segmental hypercellularity. Diffuse glomerular basement membrane (GBM) thickening was evident. Jones silver stain highlighted some foci of double‐contour formation caused by the interposition of pale material between the endothelium and the GBM. Notably, there was no segmental endocapillary proliferation, no crescents, and no glomerulitis. Congo red staining was negative, ruling out amyloid deposition. The tubulointerstitial compartment showed mild interstitial infiltration by inflammatory cells, predominantly lymphocytes, along with mild tubular atrophy and approximately 20% interstitial fibrosis. Some tubules contained intraluminal hyaline casts, and proximal tubular epithelial cells exhibited cytoplasmic resorptive droplets and simplification. Vascular examination revealed mild atherosclerosis (Figure 1).

A direct IF study was performed on frozen sections containing 3 glomeruli. All tested markers were negative, including polyvalent immunoglobulin (G.A.M.), IgG, IgA, IgM, kappa, lambda, C3c, C4c, C1q, fibrinogen, and albumin, indicating the absence of immune complex deposition.

**FIGURE 2:**
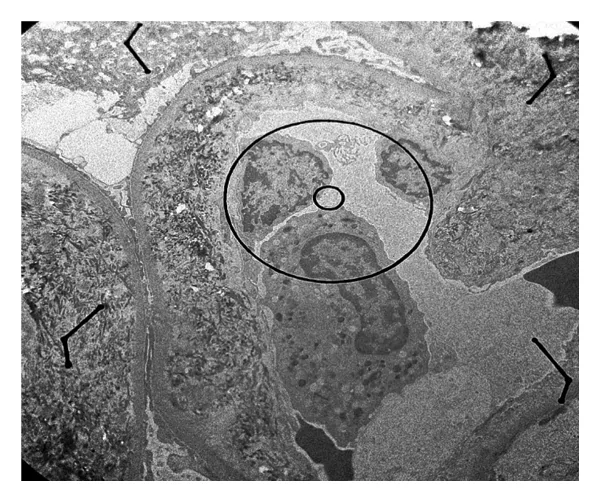
Characteristic curvilinear fibrils of collagen in the mesangium, GBM, and the subendothelial space.

EM was critical in establishing the diagnosis. EM revealed global effacement of visceral epithelial foot processes, microvillous transformation of foot processes, and vacuolization of podocytes. There was global thickening of the GBM. Most importantly, the mesangium and subendothelial space contained randomly arranged curvilinear fibrils measuring approximately 60 nm in diameter (Figure 2). No obvious subepithelial, mesangial, paramesangial, or subendothelial electron‐dense deposits were identified.

Based on these ultrastructural findings, the final diagnosis was collagenofibrotic glomerulopathy. The patient has been followed for 9 months postdiagnosis. He was treated with antihypertensive agents for blood pressure control and corticosteroid therapy. Corticosteroids are currently being tapered. His renal function is relatively stable with a recent serum creatinine of 2.3 mg/dL.

### 2.2. Case 2

A 36‐year‐old male presented with edema, markedly elevated serum creatinine, proteinuria, and hematuria. He had undergone a prior kidney biopsy that was diagnosed as focal segmental glomerulosclerosis (FSGS). Despite this diagnosis, his renal function continued to deteriorate. Laboratory evaluation at the time of presentation revealed serum creatinine of 6.75 mg/dL and blood urea nitrogen (BUN) of 51.8 mg/dL. Urinalysis showed 4+ proteinuria and 3+ hematuria. Given the discrepancy between the clinical course and the prior diagnosis, a repeat renal biopsy with EM was performed to reassess the underlying pathology.

**FIGURE 3:**
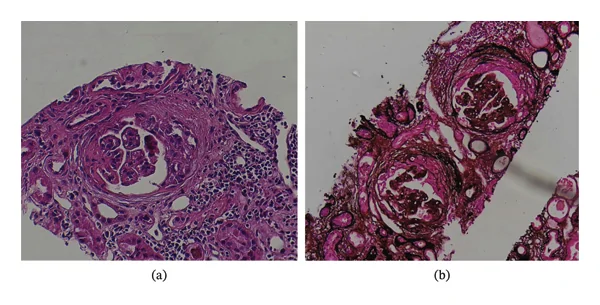
(a) Mesangial hypercellularity and interstitial inflammation in H&E stain, x800 magnification. (b) Fibrocellular crescents in Jones silver stain, x200 magnification.

Light microscopy of the repeat biopsy showed needle‐shaped corticomedullary tissue containing up to 12 glomeruli on serial sections. Most glomeruli exhibited global or segmental sclerosis. There was global mesangial matrix expansion and hypercellularity in the majority of glomeruli. Segmental GBM thickening with a double‐contour appearance was noted. Remarkably, most glomeruli showed cellular and fibrocellular crescents or pseudocrescents, and mild glomerulitis was present (Supporting Figures 1 and 2). No endocapillary proliferation was observed. The tubulointerstitial compartment demonstrated severe tubular atrophy and proportional interstitial fibrosis exceeding 50%. Mild interstitial infiltration by inflammatory cells, mainly lymphocytes, was present. Some tubules contained intraluminal hyaline casts. Vascular examination revealed mild atherosclerosis (Figure 3).

Direct IF study was performed on frozen sections containing 4 glomeruli. There was 1+ granular staining along the GBM for polyvalent immunoglobulin (G.A.M.), IgG, kappa, lambda, and C3c. IgA, IgM, C4c, C1q, and fibrinogen were all negative. Albumin showed 1+ staining in resorptive droplets within tubular epithelial cells. Thick sections containing 2 glomeruli confirmed GBM thickening and cellular crescents, along with interstitial fibrosis and tubular atrophy.

**FIGURE 4:**
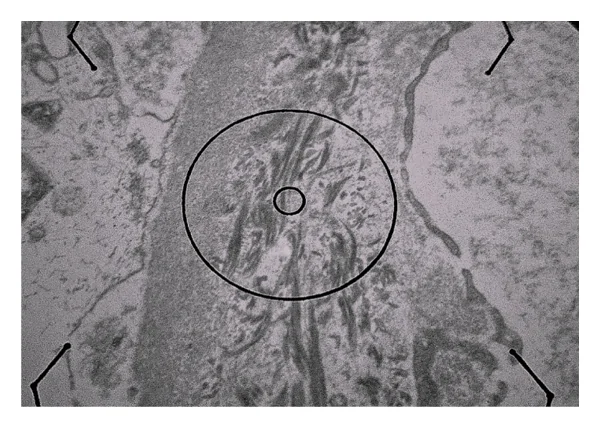
Collagen forms stacked bundles that show irregular, frayed edges and mild curvilinear morphology.

EM was decisive in establishing the correct diagnosis. EM revealed global effacement of visceral epithelial foot processes, microvillous transformation of foot processes, and vacuolization of podocytes. There was global thickening of the GBM with GBM duplication. Most strikingly, large accumulations of collagen fibrils with a frayed and curved appearance were present in the subendothelial space (Figure 4). The mesangium showed the expansion of the GBM‐like matrix material without mesangial hypercellularity. No obvious subepithelial, mesangial, paramesangial, or subendothelial electron‐dense deposits were identified.

The final diagnosis was diffuse glomerulosclerosis with severe tubular atrophy and interstitial fibrosis, with ultrastructural findings compatible with Type III collagen glomerulopathy. The patient did not respond to supportive management and developed progressive renal failure, leading to kidney transplantation 7 months after the initial biopsy. At 6 months post‐transplantation, he is presenting with mild graft dysfunction and proteinuria, which is currently being investigated.

## 3. Discussion

Type III collagen glomerulopathy, also known as collagenofibrotic glomerulopathy, is a very rare renal disease characterized by abnormal accumulation of Type III collagen fibrils within the mesangial matrix and subendothelial space of glomeruli [4, 10]. Since Type III collagen is normally restricted to the renal interstitium and vascular structures, its deposition within glomeruli represents a distinctly abnormal finding. Due to its rarity and nonspecific clinicopathologic presentation, the diagnosis may easily be overlooked, particularly when EM is not available.

The two cases presented in this report demonstrated many of the clinical features described in previously reported adult cases, including proteinuria, hematuria, hypertension, and progressive renal insufficiency [2, 4, 8]. However, both cases also illustrate one of the most important practical aspects of this disease: the difficulty of diagnosis based solely on light microscopy and IF findings. In both patients, the initial renal biopsies failed to establish the correct diagnosis. The first biopsy in Case 1 was interpreted as tubulointerstitial nephritis with acute tubular injury, while Case 2 was initially diagnosed as FSGS. Only after repeat biopsy with ultrastructural examination was the diagnosis of Type III collagen glomerulopathy established.

This diagnostic difficulty has also been emphasized in previous reports. Matthai et al. described the importance of ultrastructural evaluation in distinguishing collagenofibrotic glomerulopathy from other glomerular diseases with mesangial expansion and basement membrane remodeling [12]. Similarly, several published cases have initially been misclassified as membranoproliferative glomerulonephritis (MPGN), diabetic nephropathy, or chronic sclerosing glomerular disease because of overlapping histologic findings on light microscopy [8, 13]. Our cases further support the concept that Type III collagen glomerulopathy may remain unrecognized unless EM is specifically performed.

On light microscopy, both of our cases demonstrated findings that have been repeatedly reported in the literature, including mesangial matrix expansion, GBM thickening, and double‐contour formation [2, 4]. However, these findings are nonspecific and may mimic several chronic glomerular disorders. In Case 1, the glomeruli showed mesangial expansion by pale material with focal GBM duplication but without sclerosis or crescents, resembling a chronic MPGN‐like pattern. In contrast, Case 2 showed advanced chronic damage with global and segmental glomerulosclerosis, severe interstitial fibrosis, and extensive tubular atrophy. The advanced sclerosis in the second patient likely contributed to the initial interpretation of FSGS. EM was decisive in both of our cases. Ultrastructural examination revealed characteristic curvilinear collagen fibrils within the mesangial and subendothelial spaces, confirming the diagnosis. The fibrils measured approximately 60 nm in Case 1 and appeared as large frayed accumulations in Case 2. These ultrastructural findings are considered pathognomonic and remain the cornerstone of diagnosis [10, 14]. In addition, both patients demonstrated diffuse podocyte foot process effacement and microvillous transformation, findings that correlate with the marked proteinuria observed clinically.

The importance of EM has been consistently emphasized in the literature. According to Duggal et al., collagenofibrotic glomerulopathy showed nonspecific histologic findings, while the diagnosis became evident only after the identification of Type III collagen fibrils ultrastructurally [8]. Similarly, Kurien et al. reported that the disease may be underrecognized because its morphologic features overlap with more common glomerular pathologies [7]. Our findings align closely with these observations and further support the idea that Type III collagen glomerulopathy may be more underdiagnosed rather than truly rare in absolute frequency.

An additional notable finding in Case 2 was the presence of cellular and fibrocellular crescents or pseudocrescents. Crescent formation is uncommon in Type III collagen glomerulopathy and has only rarely been described in previous reports [13]. The presence of crescents may further complicate the histopathologic interpretation by raising concern for crescentic glomerulonephritis or other proliferative diseases. Despite these atypical features, EM clearly demonstrated the characteristic collagen fibrils required for diagnosis.

IF findings in our patients were either negative or nonspecific, which is consistent with most previously reported cases [6]. Case 1 showed complete absence of immune deposits, while Case 2 demonstrated only weak staining for IgG and C3c along the GBM. Similar nonspecific IF findings have been reported in other studies and generally do not explain the degree of glomerular injury [6, 8]. Therefore, IF alone is insufficient for diagnosis and mainly serves to exclude immune complex‐mediated diseases.

The pathogenesis of this disease remains incompletely understood. Some authors have proposed that activated mesangial cells may acquire a fibroblast‐like phenotype capable of producing Type III collagen [15], whereas others have suggested a systemic abnormality of collagen metabolism [16]. Nevertheless, most adult‐onset cases, including ours, appear to occur sporadically without a significant family history or evidence of systemic collagen deposition [2, 8]. No extrarenal manifestations were identified in either of our patients.

The last important observation in our report is the broad morphologic spectrum of the disease. Case 1 represented a relatively earlier stage with preserved glomerular architecture and mild chronic injury, whereas Case 2 showed advanced diffuse glomerulosclerosis with severe tubulointerstitial scarring. Similar variability has been described in prior studies and likely reflects the differences in disease duration and timing of biopsy [4, 12, 13]. Delayed recognition may therefore contribute to progressive irreversible renal damage before the diagnosis is established.

## 4. Conclusion

Type III collagen glomerulopathy is a rare and probably underrecognized glomerular disease with nonspecific clinical and histologic findings. Our cases demonstrate that diagnosis may be extremely difficult in the absence of EM, particularly when light microscopic features mimic more common entities such as FSGS or MPGN. The identification of characteristic collagen fibrils on ultrastructural examination remains essential for definitive diagnosis. Greater awareness of this entity among nephrologists and renal pathologists, together with appropriate use of EM in diagnostically challenging cases, is crucial to avoid misdiagnosis and improve recognition of this uncommon disorder.

## Funding

No funding was received for this manuscript.

## Conflicts of Interest

The authors declare no conflicts of interest.

## Supporting Information

Additional supporting information can be found online in the Supporting Information section.

## Supporting information


**Supporting Information** Supporting Figure 1: Fibrocellular crescent and interstitial fibrosis in trichrome stain, × 200 magnification. Supporting Figure 2: PAS stain, × 200 magnification.

## Data Availability

The data that support the findings of this study are available on request from the corresponding author. The data are not publicly available due to privacy or ethical restrictions.
