# Supplementary material for: Type III Collagen Glomerulopathy Presenting With Divergent Histopathological Patterns: Report of Two Adult Cases and Diagnostic Pitfalls
Source: Case Rep Nephrol. 2026 Sep 22;2026:6022775. doi: 10.1155/crin/6022775 (PMC13595398; doi:10.1155/crin/6022775)
Supplement: Supplementary file 1 [file CRIN-2026-6022775-s001.docx]

Supplementary Figure 1. Fibrocellular crescent and interstitial fibrosis in trichrome stain, x 200 magnification.

Supplementary Figure 2. PAS stain, x200 magnification.
